# Lifetime antimicrobial use is associated with weight status in early adolescence—A register‐based cohort study

**DOI:** 10.1111/ijpo.12727

**Published:** 2020-09-21

**Authors:** Rejane Augusta de Oliveira Figueiredo, Eero Kajantie, Pertti J. Neuvonen, Trine B. Rounge, Elisabete Weiderpass, Heli Viljakainen

**Affiliations:** ^1^ Folkhälsan Research Center Helsinki Finland; ^2^ Faculty of Medicine University of Helsinki Helsinki Finland; ^3^ National Institute for Health and Welfare Helsinki and Oulu Finland; ^4^ Children's Hospital Helsinki University Hospital and University of Helsinki Finland; ^5^ PEDEGO Research Unit MRC Oulu, Oulu University Hospital and University of Oulu Finland; ^6^ Department of Clinical and Molecular Medicine Norwegian University of Science and Technology Trondheim Norway; ^7^ Department of Clinical Pharmacology University of Helsinki and Helsinki University Hospital Helsinki Finland; ^8^ Department of Research Cancer Registry of Norway Oslo Norway; ^9^ Department of Informatics University of Oslo Oslo Norway; ^10^ International Agency for Research on Cancer World Health Organization Lyon France; ^11^ Department of Food and Nutrition University of Helsinki Helsinki Finland

**Keywords:** adolescents, antimicrobials, children, overweight, register study, weight status

## Abstract

**Background:**

Antimicrobial agents (AMs) are the most prescribed drugs to children. Early and repeated exposure to AMs in infancy is associated with increased risk of childhood overweight and obesity.

**Aims:**

We extended the investigation of AMs use, from birth to early adolescence, and evaluated their association with weight status.

**Materials & Methods:**

A total of 10093 children from Finnish Health in Teens cohort (Fin‐HIT) with register‐based data on AMs purchases and measured weight status at the mean age of 11.2 y (SD 0.82) were included in the study. The key exposures were the number AM purchases at a given age or the sum of these during the entire follow‐up time to describe lifetime exposure / use. Outcome was weight status in early adolescence defined with International Obesity Task Force cut‐offs for the age‐ and sex‐specific body mass index. Odds Ratio (OR) and 95% confidence intervals (CI) were estimated using Multinomial Logistic Regression.

**Results:**

Of children, 73.7% were normal weight, 11.1% thin and 15.2% overweight/obese. AMs use was highest during the second year of life, when 65% of all children used AMs, but thereafter decreased with age. The highest mean purchases and prevalence at any given age along with the highest lifetime use were consistently seen among overweight children. Each episode of AMs use throughout life increased the risk of being overweight in adolescence [OR = 1.02 (1.02–1.03)]. However, there was an inverse association between AMs use and thinness [OR = 0.98 (0.97–0.99)].

**Discussion:**

Despite a high prevalence of AMs use during the early years, lifetime‐use was associated with weight status in early adolescence in a dose response manner.

**Conclusion:**

Future studies should address mechanisms underlying the relationship between AM use and weight.

## 1 INTRODUCTION

Elderly adults (≥60 years) and children up to 9 years old are exposed to the highest amount of antimicrobial agents (AMs).^1^ In many countries, AMs are among of the most prescribed drugs in young children and adolescents,^2^, ^3^, ^4^ especially in the early stage of life.^5^ For instance, 40% of Danish children are prescribed AM during the first year of life.^6^ In many low‐ and middle‐income countries, AMs are frequently purchased over‐the‐counter, which may increase the risk of inappropriate use.^7^ Moreover, in low‐resource settings, there is a considerable research interest to study mass antibiotic treatment to pregnant women or children in order to reduce child mortality and improve growth and development.^8^, ^9^

Repeated perturbations caused by the use of AMs are likely to shift the microbiota's community structure by eliminating some strains and allowing new strains to enter the community. This may affect the microbiome's capacity and host metabolism.^10^, ^11^ It is suggested that AM‐induced microbiome alterations may increase susceptibility to intestinal infections, immune‐mediated diseases and dysregulation of metabolism, likely contributing to weight gain especially in children.^10^ Overweight and obesity are global health problems and, to date, in Finland, the combined prevalence in children is around 15%.^12^, ^13^ The contribution of accumulated lifetime AM use for the obesity epidemic is yet to be determined.

In 2015, World Health Organization (WHO) introduced a global action plan to improve the quality of AM use and to decrease the number of unnecessary prescriptions.^14^ In European countries, where national actions to improve the rational use of antibiotics have been applied, a decreasing trend in AM use has been reported, likewise in Finland.^4^ Still differences in AM use between countries appear: in 2018, the mean total consumption of AMs for systemic use in community and hospital sector was 15.5 defined daily doses per 1000 inhabitants per day (DDD) in Finland, while the mean was 20.1 DDD in the EU/EEA, ranging from 9.7 in the Netherlands to 34 in Greece.^4^

The primary purpose of the present study was to evaluate if an exposure to AMs at any given age, or the total/lifetime exposure from birth to early adolescence, is associated with weight status at early adolescence. As a secondary aim, the trends in AM use and exposure during pregnancy were described and their relevance to weight status were evaluated. Previously, early and repeated exposure to AMs in infancy have been associated with increased risk of childhood overweight and obesity.^14^, ^15^, ^16^ In several studies, the follow‐up times have been relatively short^15^, ^17^, ^18^ and they have paid less or no attention to accumulated exposure. We extended the investigations by utilizing the nationwide data on AM purchases from well‐defined Finnish national registries as an objective measure of lifetime AMs exposure^19^ and evaluated its association with weight status in early adolescence.

## 2 METHODS

This study was conducted among participants of the Finnish Health in Teens cohort (Fin‐HIT), which is a school‐based cohort of mainly 9 to 12 years old children without specific exclusion criteria. In total, 11 407 children and one of their parents (n = 9935, mostly mothers) participated the baseline study in 2011 to 2014. Details on the Fin‐HIT cohort are described elsewhere.^13^ The Coordinating Ethics Committee of the Hospital District of Helsinki and Uusimaa has approved the study protocol (169/13/03/00/10) including linkage to national health registers, and written informed consent was obtained from all participants and their parents.

### 2.1 Variables in the study

Age in years, sex and language spoken at home (Finnish, Swedish and others) for children and mothers were obtained from the consent form and were confirmed by record linkages with the National Population Information System at the Population Register Centre. Mothers’ anthropometric measures and educational level were obtained using a questionnaire at baseline data collection. Children's height (in m) and weight (in kg) were measured in a standardized way by trained field workers. Children's BMI (weight in kg divided by height in m^2^) to describe weight status was categorized as thin, normal weight, overweight and obese according to the International Obesity Task Force.^20^ However, we combined children with overweight and obesity (denoted as overweight) in the analyses because the number of children with obesity was small (2.4%). Not having weight status is an exclusion criterion for the sample size in the present study.

Maternal education level (n = 4303) was dichotomized into high (≥UNESCO ISCED category 6) or low (≤ISCED 5) education.^21^

### 2.2 Register data on antimicrobials

Children's and mothers’ information on AM purchases were obtained from the Drug Prescription Register maintained by the Social Insurance Institution of Finland (KELA). The data utilizes Anatomical Therapeutic Chemical classification system held by WHO,^22^ and date of each purchase. The registry does not include medicines that are not reimbursable nor medicines that are inexpensive and cost less than the set reimbursement level.^19^ The linkage to the register data has been made through the participants’ unique social security numbers. Medications prescribed for the child are registered with his/her social security number, and those prescribed to the mother are registered with the mother's social security number. Medical purchase history was available from 11 212 children and 7508 mothers from January 1, 1996 to December 31, 2014. In our study, we selected all AMs for systemic use (J01) and directly acting antivirals (J05), but not antimycotics (J02) or antimycobacterials (J04) as these are not directly affecting human microbiota. The following classification was used: tetracyclines (J01AA), penicillins with extended spectrum (J01CA), beta‐lactamase sensitive penicillins (J01CE), combinations of penicillins, including beta‐lactamase inhibitors (J01CR), cephalosporins first generation (J01DB), cephalosporins second generation (J01DC), cephalosporins third generation (J01DD), sulfonamide and trimethoprim (J01E), macrolides (J01FA), lincosamides (J01FF), fluoroquinoles (J01MA), other antibacterials (J01X) and antivirals (J05).

Date of purchase is considered to reflect the use of AM. Together with children's birth date, we created following variables: for each participant, we summarized AM use for the first 6 months, for each full year of age, and total use from birth to early adolescence (eg, here referred as lifetime use) as main exposures. At each full year of age, the number of AM purchases was dichotomized (1/0) to describe the prevalence of AM use. Mothers’ AM use during pregnancy reflecting prenatal exposure was studied as a secondary aim, while postnatal maternal AM use was considered as a confounder. Other medical purchases excluding AMs were summarized and used as a proxy of overall morbidity of the child in further analyses.

Data on Medical Birth Registry held by the National Institute for Health and Welfare were used to confirm that the children were born in Finland (n = 10 944) and to exclude extremely preterm born children (<28 weeks, n = 12), as these might affect the results. Thus, records were considered for 10 772 children and 7220 mothers (Figure S1).

### 2.3 Statistical analysis

Categorical variables between weight status were compared using chi‐square tests. Continuous variables were compared between two groups using *t*‐test. Comparisons of continuous variables between more than two groups were carried out using ANOVA, and it was adjusted by Brown‐Forsythe test when appropriate. Prevalence of AMs at different ages were compared between weight status using chi‐square test, and residual analysis was carried out to identify differences between the categories. In these analyses, Bonferroni correction was used to adjust for multiple tests. Association between variables related to the use of AM and the children's weight status was estimated through odds ratio (OR) and 95% confidence intervals (95% CI) by multinomial logistic regression, as weight status was evaluated in three categories. Multivariate models were created for children with information on weight status (ie, BMI, gender and age) (n = 10 093) and were adjusted for age, gender, language spoken at home and number of medical purchases other than AMs (Figure S1). When mother's use of AM during pregnancy was included, models were estimated for those who had mother's information (n = 6708) available and were adjusted for age, gender, language spoken at home and mother's age.

All statistical analyses were carried out using SPSS version 25, and a 5% statistical significance level was adopted.

## 3 RESULTS

The average age for children in baseline study visit was 11.2 years (SD = 0.86) and for mothers 41.7 (5.3) years. Of the children, 52.5% were girls. Most children were normal weight (73.7%), while 11.1% were thin and 15.2% were overweight. Of the mothers, 57.9% were normal weight and about 40% overweight or obese (Table 1). Information on educational level was available for 4303 mothers: 53.7% of them had a high educational level and 46.3% had a low educational level. In Table 1, we present the number of AM purchases for children and mothers compared between several participant's characteristics. These unadjusted results suggest a higher total number of AM purchases for boys, overweight children and children who used AMs during the first 6 months of life. Mothers who used AMs during pregnancy or during the first 6 months of child's life also had a higher number of AM purchases during the follow‐up time compared with mothers who had not used AMs during these periods. Furthermore, their children used more AMs than children to mothers without consecutive use. There was a 28% less AM purchase for children whose families use languages other than Finnish or Swedish at home, likely indicating a migrant background, when compared to children of families using Finnish or Swedish.

**TABLE 1:** General description of the participants and total purchases by baseline characteristics collected in 2011 to 2014

		Children (n = 10 093)		Total Purchases—Children		*P*‐value	Mothers (n = 6708)		Total Purchases—Mothers		*P*‐value
		n	%	Mean	SD		n	%	Mean	SD	
Child’s gender	girl	5295	52.50%	10.01	8.32	<0.001	3522	52.50%	10.18	8.92	0.002
	boy	4798	47.50%	11.24	8.7		3186	47.50%	10.87	9.64	
Child’s weight status	thin	1119	11.10%	9.0 0	6.99	<0.001 (BF)	719	10.80%	9.93	9.78	<0.001 (BF)
	normal weight	7434	73.70%	10.43	8.37		4955	73.90%	10.31	9.08	
	overweight	1540	15.20%	12.55	9.86		1034	15.30%	11.87	9.72	
Language spoken at home	Finnish	9543	94.60%	10.66	8.57	<0.001 (BF)	6381	95.10%	10.57	9.3	0.025 (BF)
	Swedish	423	4.20%	9.98	7.84		284	4.20%	9.73	8.82	
	others	127	1.20%	7.51	6.53		43	0.70%	7.58	8.41	
Child’s AM use until 6 months	no	8167	80.90%	9.98	7.7	<0.001	5436	81.00%	10.07	8.76	<0.001
	yes	1926	19.10%	14.7	9.98		1272	19.00%	12.38	11.03	
Mother’s education level^a^	low	1994	46.30%	10.94	8.64	0.036	1989	46.30%	10.79	9.66	<0.001
	high	2309	53.70%	10.4	8.12		2305	53.70%	9.71	8.19	
Mother’s weight status^b^	thin			10.68	7.28	<0.001 (BF)	74	1.70%	12.39	12.84	<0.001 (BF)
	normal weight			10.35	8.26		2527	58.10%	9.6	8.5	
	overweight			10.64	8.03		1182	27.20%	10.2	8.51	
	obese			11.93	9.31		566	13.00%	12.94	10.5	
Mother’s AM use during pregnancy^c^	no	4314	64.30%	10.12	8.3	<0.001	4314	64.30%	8.41	7.32	<0.001
	yes	2394	35.70%	11.94	8.72		2394	35.70%	14.29	11.06	
Mother’s AM use until 6 months of child’s life ^c^	no	4678	69.70%	10.21	8.23	<0.001	4314	64.30%	8.41	7.32	<0.001
	yes	2030	29.30%	12.11	8.94		2394	35.70%	14.29	11.06	

AM; antimicrobial, SD; standard deviation, BF; Brown‐Forsythe

^a^ n=4303

^b^ n=4349

^c^ n=6708

From 1998 to 2014, of all children (97.9%) had an AM purchase recorded, and of all medicine purchases AMs accounted for 66% among children and 22.3% for mothers. In the same period, the total number of AM purchases varied between 0 and 143, both in children and their mothers, while the mean number of AM prescriptions was 10.6 (8.6) per child and 10.4 (9.4) per mother.

the prevalence of AMs use by full year of age amongst children; the prevalence of AMs use during the first year was 46% and during the second year (full year of age 1) 65%, which was the highest prevalence in all ages. After age 2, the prevalence decreased with age.

**FIGURE 1:**
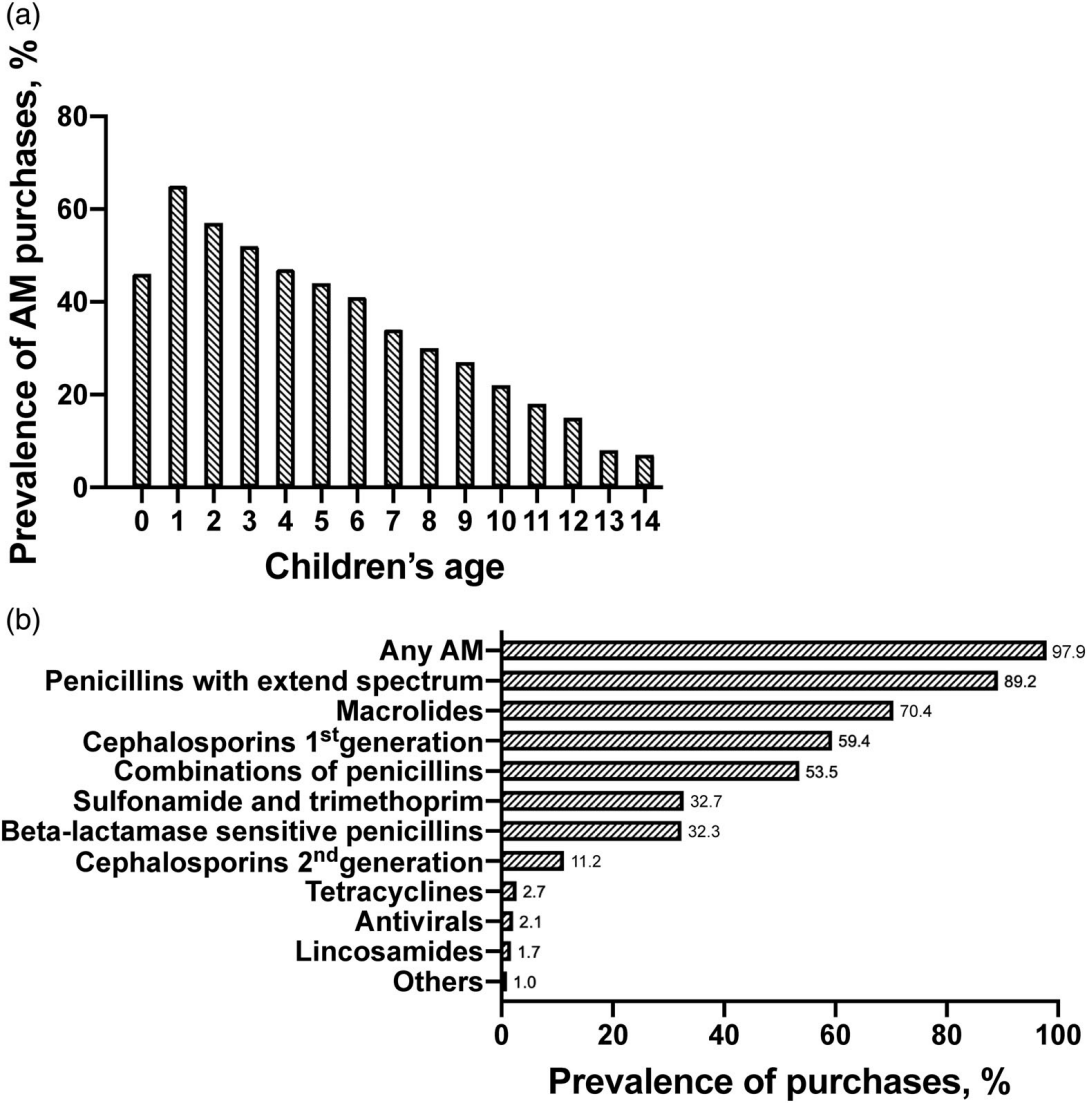
Prevalence of antimicrobial purchases (%) by age (A) and type (B). Others include fluoroquinoles, other antibacterials and Cephalosporins third generation

**FIGURE 2:**
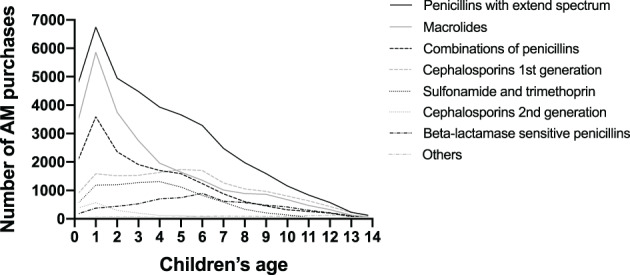
Number of purchases by children's age separated by type of antimicrobial. Others represent the sum of Cephalosporins third generation, lincosamides, antivirals, other antibacterials, tetracyclines and floroquinoles

The AM with the highest prevalence throughout the follow‐up time was penicillin with extended‐spectrum (89.2%), followed by macrolides (70.4%), first generation cephalosporins (59.4%) and combinations of penicillins, including beta‐lactamase inhibitors (53.5%) (Figure 1B). Figure 2 shows that the penicillins with extended spectrum, macrolides, and combinations of penicillins were the most commonly prescribed AMs during the first years of life. Penicillin with extended spectrum had the highest frequency of use up to 13 years of age.

**FIGURE 3:**
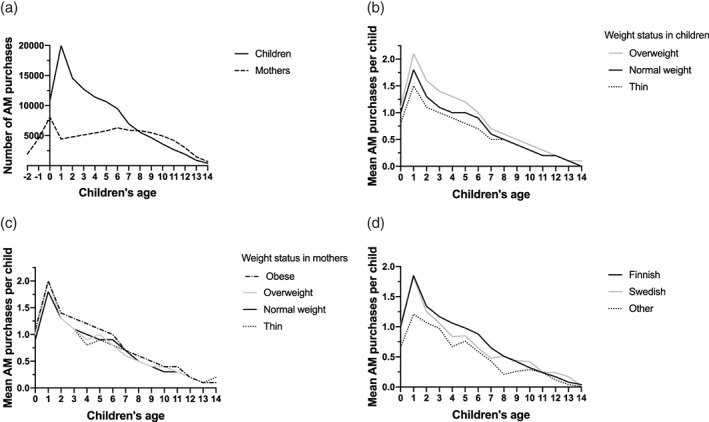
Total antimicrobial purchases in mothers and children (A); mean antimicrobial purchases by children's weight status (B); by mothers’ weight status (C); and by home language (D)

We observed no association between maternal and child AM purchases during the follow‐up time (Figure 3A). Children with overweight had a higher number of AM purchases compared with other weight status at least until 12 years of age (Figure 3B), while no differences in children's AM purchases were observed by mothers’ weight status (Figure 3C). Interestingly, the number of purchases for children with foreign home language was lower especially during early childhood compared with Finnish and Swedish speaking children (Figure 3D).

The prevalence of AM purchases was compared between children's weight status (Table S1): higher prevalence in overweight children and lower prevalence in thin children are observed in most ages. In Table 2, we further compared baseline characteristics between children's weight status. The higher the weight status, the higher percentage of children and mothers used AMs during the first 6 months of child's life. In addition, the first exposure to AMs occurred at earlier stage in overweight group than with other weight status. On the other hand, mother's AMs use during pregnancy was not associated with child's weight status.

**TABLE 2:** Baseline characteristics and total purchases by children's weight status

		Thin 11.1%(n = 1119)		Normal 73.7%(n = 7434)		Overweight 15.2%(n = 1540)		*P*‐value
		n	%	n	%	n	%	
**General Characteristics**								
Child’s gender	girl	682	60.90%	3839	51.60%	774	50.30%	<0.001
	boy	437	39.10%	3595	48.40%	766	49.70%	
Language spoken home	Finnish	1066	95.30%	7016	94.40%	1461	94.90%	0.038
	Swedish	35	3.10%	334	4.50%	54	3.50%	
	others	18	1.60%	84	1.10%	25	1.60%	
**AM purchases in children**								
Child’s AM use until 6 months	(yes)	172	15.50%	1430	19.40%	324	21.20%	0.001
First AM before 2 years old	(yes)	310	27.90%	1714	23.20%	330	21.60%	0.001
Mother’s AM use during pregnancy	(yes)	511	71.10%	3478	70.20%	689	66.60%	0.135
Mother’s AM use until 6 months of child’s life	(yes)	803	72.10%	5673	76.80%	1198	78.40%	0.04
		Mean	SD	Mean	SD	Mean	SD	
**Age**								
Child, y		11.2	0.9	11.1	0.8	11.1	0.8	0.008
Mother, y		41.7	5.3	41.7	5.3	41.8	5.4	0.883
**Number of purchases**								
**Children**								
Total purchases		8.84	6.95	10.26	8.34	12.3	9.88	<0.001 (BF)
Total purchases by type								
Penicillin with extend spectrum		3.22	2.74	3.64	3.41	4.29	3.4	<0.001 (BF)
Combinations of penicillins		1.32	2.32	1.55	2.48	1.8	3.23	<0.001 (BF)
Cephalosporins 1^st^ generation		1.84	2.37	2.19	2.74	2.68	3.48	<0.001 (BF)
Macrolides		1.16	1.54	1.42	2	1.71	2.27	<0.001 (BF)
Child's age at 1^st^ AM course		1.27	1.82	1.09	1.75	1.02	1.7	0.001
Number of purchases until <6 months		1.48	0.92	1.46	0.85	1.51	0.88	0.62
Number of purchases until <1 year old		0.83	1.42	0.99	1.45	1.11	1.56	<0.001 (BF)
Number of purchases until <2 years old		2.37	2.72	2.83	2.91	3.19	3.23	<0.001 (BF)
**Mothers (n=6708)**								
Number of purchases during pregnancy		1.38	0.8	1.5	0.94	1.62	1.08	0.007
Total purchases		9.95	9.78	10.3	9.07	11.86	9.76	<0.001

### 3.1 Multinomial regression models

Table 3 shows the results for several multivariate models evaluated in this study. In all models, the total number of AM purchases was positively associated with overweight and inversely associated with thinness (*P* < 0.05). We found that children's AMs use during the first 6 months (OR = 0.80; 95% CI: 0.67‐0.95) and when they have completed 2 years (OR = 0.83; 95% CI: 0.72‐0.96) were inversely associated with thinness. However, these variables did not remain statistically significant in the models after adjusting for total number of prescriptions (models 5 and 6), pointing out that the total number of prescriptions had a greater impact on BMI than the early use. Furthermore, the association of lifetime AM purchases with preadolescent's BMI was not affected by intrauterine exposure to AM. In the models evaluating number of purchases separately for the four main AM types, we observed that penicillin with extended spectrum and first generation cephalosporins were consistently positively associated with overweight and inversely associated with thinness (*P* < 0.05). This was not the case for macrolides.

**TABLE 3:** Odds ratio (OR) and 95% confidence intervals (CI) for children’s weight status

Model	Principal variables in the model	Thinness				Overweight			
		p‐value	OR	95% CI		p‐value	OR	95% CI	
				Lower	Upper			Lower	Upper
**Only child information** ^**a**^ **(n=10093)**									
1	Total AM	**<0.001**	0.98	0.97	0.99	**<0.001**	1.02	1.02	1.03
2	Children used AM ‐ until 6 months	**0.012**	0.8	0.67	0.95	0.183	1.1	0.96	1.26
3	Children used AM before 2 years old	**0.013**	0.83	0.72	0.96	0.293	1.08	0.94	1.23
4	Age at 1^st^ AM	**0.021**	1.04	1.01	1.08	0.287	0.98	0.95	1.02
5	Total AM	**<0.001**	0.98	0.97	0.99	**<0.001**	1.03	1.02	1.03
	Children used AM ‐ until 6 months	0.124	0.87	0.73	1.04	0.709	0.97	0.85	1.12
6	Total AM	**<0.001**	0.98	0.97	0.99	**<0.001**	1.03	1.02	1.03
	Children used AM before 2 years old	0.4	0.94	0.8	1.09	0.169	0.91	0.79	1.04
7 ^c^ (n=9814)	Total AM	**<0.001**	0.98	0.97	0.98	**<0.001**	1.03	1.02	1.04
	Age at 1^st^ AM	0.501	1.01	0.98	1.05	0.154	1.03	0.99	1.06
**Including mother’s information** ^**b**^ **(n=6708)**									
8	Total AM	**<0.001**	0.98	0.97	0.99	**<0.001**	1.02	1.01	1.03
	Children used AM ‐ until 6 months	0.761	1.03	0.83	1.28	0.886	1.01	0.85	1.2
	Mothers used AM during pregnancy	0.687	0.97	0.82	1.14	0.462	1.05	0.92	1.21
9	Total AM	**<0.001**	0.98	0.97	0.99	**<0.001**	1.02	1.02	1.03
	Children used AM before 2 years old	0.488	0.94	0.77	1.13	0.493	0.94	0.79	1.12
	Mothers used AM during pregnancy	0.711	0.97	0.82	1.15	0.442	1.06	0.92	1.22
10 ^c^ (n=6542)	Total AM	**<0.001**	0.98	0.97	0.99	**<0.001**	1.02	1.02	1.03
	Age at 1^st^ AM	0.715	1.01	0.97	1.05	0.631	1.01	0.97	1.06
	Mothers used AM during pregnancy	0.81	0.98	0.83	1.16	0.494	1.05	0.91	1.21
**Separated by type AM (n=10093)** ^**a**^									
11	Total penicillins with extend spectrum	**0.005**	0.97	0.94	0.99	**<0.001**	1.04	1.02	1.06
	Total combinations of penicillins	0.997	1	0.97	1.03	0.849	1	0.98	1.02
	Total cephalosporins 1^st^ generation	**0.005**	0.94	0.91	0.98	**0.002**	1.04	1.01	1.07
	Total macrolides	0.136	0.98	0.95	1.01	**0.002**	1.02	1.01	1.05
	Children used AM ‐ until 6 months	0.142	0.88	0.73	1.05	0.796	0.98	0.85	1.13
12	Total penicillins with extend spectrum	**0.003**	0.96	0.94	0.99	**<0.001**	1.04	1.03	1.06
	Total combinations of penicillins	0.947	1	0.97	1.03	0.786	1	0.98	1.03
	Total cephalosporins 1^st^ generation	**0.005**	0.94	0.91	0.98	**0.002**	1.04	1.02	1.07
	Total macrolides	0.127	0.98	0.95	1	**<0.001**	1.04	1.01	1.06
	Children used AM before 2 years old	0.495	0.95	0.81	1.11	0.16	0.9	0.78	1.04
13 ^c^ (n=9814)	Total penicillins with extend spectrum	**0.004**	0.97	0.94	0.99	**<0.001**	1.05	1.03	1.06
	Total combinations of penicillins	0.925	1	0.97	1.03	0.753	1	0.98	1.03
	Total cephalosporins 1^st^ generation	**0.004**	0.94	0.9	0.98	**0.002**	1.04	1.02	1.07
	Total macrolides	0.114	0.98	0.95	1	**<0.001**	1.04	1.01	1.06
	Age at 1^st^ AM	0.623	1	0.97	1.05	0.139	1.03	0.99	1.06

*Note*: The bold values represent *p* < 0.05.

^a^ model adjusted by children age, gender, language spoken at home, and number of medical purchases other than AMs

^b^ model adjusted by children age, gender and language spoken at home, mother's age, and number of medical purchases other than AMs

^c^ model only with children who have used AM

## 4 DISCUSSION

In this cohort study with registry‐based medical purchase history, the highest prevalence of AMs use occurred during the first 2 years of life and thereafter decreased with age in children. This is the most extensive report on the association between AM exposure from the fetal period to early adolescence and weight status in Finnish children. We observed that higher lifetime use of AM is positively associated with overweight and inversely associated with thinness in early adolescence. Lifetime AM use overruled the effects prenatal and early‐life exposures on weight status.

Antimicrobials are widely prescribed drugs in the pediatric populations.^4^ In our study, only 2.9% of participants were considered antibiotic naïve based on the purchase registry. Of all drug purchases from 1998 to 2014 among the Fin‐HIT children, 66% were AMs related, a higher amount than reported in Denmark from 2004 to 2012 (46%).^4^ This was expected since the prevalence of AMs use has been decreasing over the years, and the evaluated period in Denmark was somewhat more recent.^4^, ^23^ Here, 46% of children used AMs during the first year of life. Comparing with other European countries in 2005 to 2007 this prevalence was slightly higher,^6^, ^24^ but again our results regarding the first years of life are for the period 1999 to 2004 when the concern on appropriate AMs use was not as widely discussed.

Previous studies have reported that the use of AMs during the first 6 months and the first 2 years of life have long‐term effects on weight gain and obesity in children^25^, ^26^, ^27^ and our findings are in line with these results. However, we uncovered that the total AM use until age 14, which is considered as lifetime AM use in this context, was even more important predictor of overweight in adolescents than the early use. Similar results were reported by Poulsen et al amongst 3‐year‐old children in the United States.^15^ Azad et al (2017) suggested that the mechanism behind weight gain is related to microbiota, implying that the use of AMs may disrupt the gut microbiota development occurring within the first 2 years of life and cause some functional alterations.^27^ AM‐induced microbiome alterations have been linked to dysregulation of metabolism, for example inducing low‐grade inflammation, decreasing insulin sensitivity and altering short‐chain fatty acid (SCFA) and bile acid metabolism,^10^ which are also present in obesity.

Penicillin with extended spectrum had the highest frequency of use up to 14 years of age. These AMs including ampicillin and amoxicillin have high efficacy against various gram‐negative bacteria. A high frequency of use of broader spectrum AMs, especially macrolides and cephalosporins, was also found. The macrolides are widely used AMs in children and adults.^15^, ^28^ The major members of this class, that is, azithromycin, erythromycin and clarithromycin have good antimicrobial activity and tissue penetration, and they mainly affect the gram‐positive pathogen.^29^ Due to increase in global resistance to penicillin and macrolide at the turn of the 21st century, the use of extended‐spectrum penicillin is recommended prior to macrolides.^30^ In our cohort, the first generation cephalosporins (59.4%) are the third most commonly used AMs, despite their reported hypersensitivity in children.^31^ The first generation cephalosporins are known to have greater activity against gram‐positive than gram‐negative bacteria, and these AMs are mainly used for skin and soft tissue infections.

Our study adds to the growing body of evidence that the use of AMs during childhood increases the risk of overweight and decreases the risk for thinness in early adolescence, while prenatal AM use does not associate with the risk of the child being overweight. While our study cannot in itself establish causation, we found evidence that the use of penicillin with extended‐spectrum and first generation cephalosporins presented independent and similar ORs as the lifetime AM use and might contribute to weight gain in early adolescence. These are the first and third most commonly purchased AMs for children in Finland, similarly to Denmark^6^ and various other countries in Europe.^32^ However, formal mediation analyses proving evidence that penicillin with extended‐spectrum and first generation cephalosporins, either together or separately, are driving the effect of lifetime AMs use on weight status were not conducted. Poulsen reported macrolides to associate most strongly with BMI z‐score among 3‐year‐old US children,^15^ whereas in the present study, their effect was similar or lower than that of penicillin with extended‐spectrum and first generation cephalosporins. Conversely, the inverse association with thinness was not consistent with macrolides. The children speaking other languages than Finnish or Swedish at home (1.2% of our study population, in total 127 children), possibly indicating migrant background, had a 28% lower AM purchases in our study. These children were born in Finland, and the most striking difference in AM purchases was noted during the two first year of life, which deserves to be explored in future studies.

The main strength of our study is its large data size with a long follow‐up time: we have collected AM use from the national Drug Prescription Register from birth to early adolescence for 10 000 children in Finland. The records were available for 98% of the participants, and these were linked with measured BMI at the mean age of 11 years, which gives us a strong, objective data on the exposure and outcome. These we have supplemented with data from Medical Birth Registry which allowed to limit the analyses to children born in Finland, and exclude those with extremely preterm birth, and with comprehensive background information supporting and allowing more complete modelling. Unfortunately, we do not have data on the disease or indication for AMs use, but since it is prescription‐based and assumed rational in terms of dosing and duration. It has been speculated, for example, ^18^ that the association between high AMs use and overweight might reflect a higher tendency for people with overweight to seek for medical care. We assumed that the sum of other medical purchases might mirror overall morbidity, and those with high number of other medications may have a chronic disease or seek medical care more often, which we wanted to consider in the analysis. However, this was not the case, since the associations remained at the same level. One of the study limitations is that we lacked information on whether AMs were taken as prescribed as we rely solely on purchase data. In addition, AMs received as inpatients were not included in the study. For this reason, we excluded extremely preterm‐born participants, who may have received a number of AM courses as newborn inpatients.^33^ On the other hand, self‐medication or over‐the‐counter sale of AMs are not issues in Finland, as AMs are prescription‐based and reimbursed drugs have relatively tight control. Our results may be extrapolated to other affluent countries, where maternal educational level is relatively high, and the use of AMs is determined by prescription given by a medical doctor.

The peak prevalence in AMs use was observed during the second year of life and decreased thereafter. Despite the high prevalence of AM use during the early years, lifetime use was the strongest determinant of weight status and it was associated with weight status in early adolescence in a dose‐response manner. Future studies should address mechanisms underlying the relationship between AM use and weight.

## CONFLICT OF INTEREST

The authors have nothing to declare.

## AUTHOR CONTRIBUTIONS

All authors contributed to study design and data collection, RF and HV carried out the literature search, data analysis and drafted the manuscript, all authors participated in data interpretation, revised the manuscript and approved the final version of the manuscript.

## Supporting information


**Figure S1** Flow chart on study population
**Table S1** Prevalence of AM use at different ages and separated by children's weight status at baseline
