# Supplementary material for: Lifetime antimicrobial use is associated with weight status in early adolescence—A register‐based cohort study
Source: Pediatr Obes. 2020 Sep 21;16(3):e12727. doi: 10.1111/ijpo.12727 (PMC7900955; doi:10.1111/ijpo.12727)
Supplement: Supplementary file 1 — Figure S1 Flow chart on study population Table S1 Prevalence of AM use at different ages and separated by children's weight status at baseline [file IJPO-16-e12727-s001.pdf]

TITLE: Lifetime antimicrobial use is associated with weight status in early adolescence – a register-based cohort study

Running head: Lifetime AM use and weight status

Authors:
Rejane Augusta de Oliveira Figueiredo [1,2]
Eero Kajantie [3, 4, 5, 6]
Pertti J. Neuvonen [7]
Trine B Rounge [1, 8, 9]
Elisabete Weiderpass [10]
Heli Viljakainen [1,11]

Affiliation:
1 Folkhälsan Research Center, Helsinki, Finland
2 Faculty of Medicine, University of Helsinki, Helsinki, Finland
3 National Institute for Health and Welfare, Helsinki and Oulu, Finland
4 Children's Hospital, Helsinki University Hospital and University of Helsinki, Finland
5 PEDEGO Research Unit, MRC Oulu, Oulu University Hospital and University of Oulu, Finland
6 Department of Clinical and Molecular Medicine, Norwegian University of Science and Technology, Trondheim, Norway
7 Department of Clinical Pharmacology, University of Helsinki and Helsinki University Hospital, Helsinki, Finland
8 Department of Research, Cancer Registry of Norway, Oslo, Norway
9 Department of Informatics, University of Oslo, Oslo, Norway
10 International Agency for Research on Cancer, World Health Organization, Lyon, France
11 Department of Food and Nutrition, University of Helsinki, Helsinki, Finland

Corresponding author:
Heli Viljakainen, Folkhälsan Research Center, Topeliuksenkatu 20, 00250 Helsinki, Finland, E-mail address: heli.viljakainen@helsinki.fi, Phone: +358 5916998.

Figure S1. Flow chart on study population.

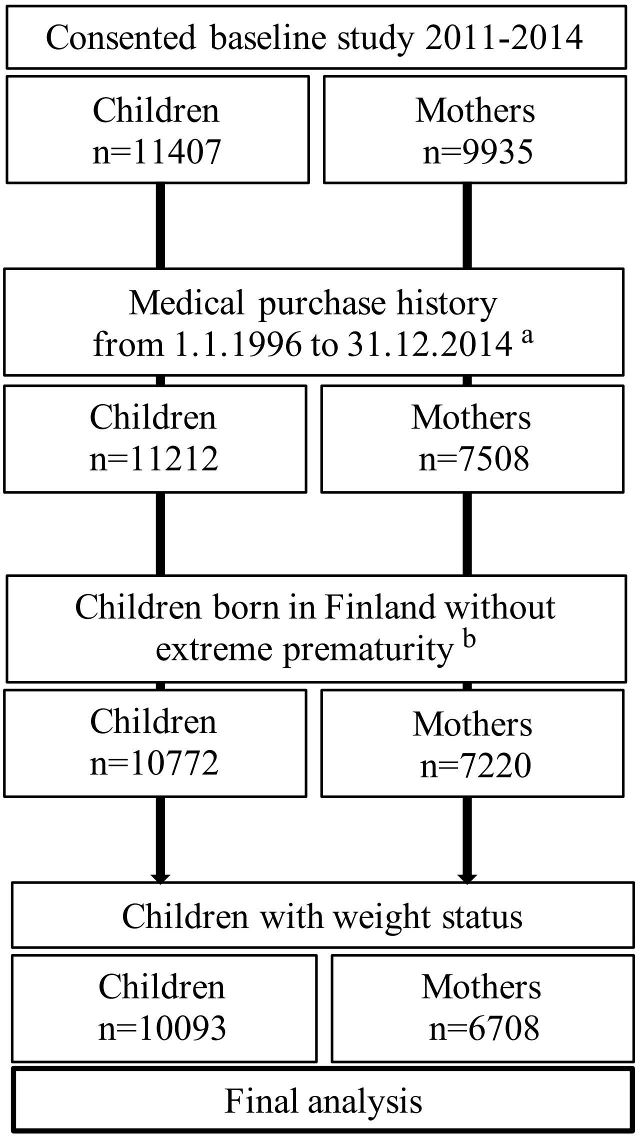

Based on the linkage to:
[a] Drug Prescription Register
[b] Medical Birth Register

Table S1. Prevalence of AM use at different ages and separated by children's weight status at baseline

Age	Thin [#]		Normal [#]		Overweight [#]		p-value *
	n	%	n	%	n	%	
until 6 months	172	15.5 % [a]	1430	19.4 %	324	21.2 % [a]	0.001
less than 1 year	442	39.7 % [a]	3510	47.5 %	761	49.8 % [a]	< 0.001
1 complete year	691	62.1 % [a]	4932	66.8 %	1073	70.2 % [a]	< 0.001
2 complete years	588	52.8 % [a]	4278	57.9 %	970	63.5 % [a]	< 0.001
3 complete years	534	48.0 %	3860	52.3 %	894	58.5 % [a]	< 0.001
4 complete years	513	46.1 % [a]	3492	47.3 %	803	52.6 % [a]	< 0.001
5 complete years	461	41.4 % [a]	3307	44.8 %	749	49.0 % [a]	< 0.001
6 complete years	413	37.1 % [a]	2992	40.5 %	716	46.9 % [a]	< 0.001
7 complete years	318	28.6 % [a]	2515	34.0 %	611	40.0 % [a]	< 0.001
8 complete years	303	27.2 % [a]	2183	29.6 %	552	36.1 % [a]	< 0.001
9 complete years	289	26.0 %	1946	26.3 %	495	32.4 % [a]	< 0.001
10 complete years	205	18.4 % [a]	1565	21.2 %	379	24.8 % [a]	< 0.001
11 complete years	171	16.7 %	1175	17.3 %	293	20.8 % [a]	0.006
12 complete years	126	13.8 %	858	14.5 %	231	18.8 % [a]	< 0.001
13 complete years	54	6.8 %	386	7.5 %	109	10.1 % [a]	0.007
14 complete years	27	6.5 %	173	6.6 %	47	8.5 %	0.276

[a] Difference identified using residual analysis

[#] Categories are based on IOTF defined age- and sex-specific BMI cut-offs

*α-adjusted < 0.0031 (significance level after Bonferroni correction).
